# *Weizmannia coagulans* BC99 Ameliorates Obesity and Associated Inflammation by Remodeling the Gut Microbiota and Regulating Lysophosphatidylcholine and Conjugated Bile Acid Metabolism

**DOI:** 10.3390/metabo16040228

**Published:** 2026-03-30

**Authors:** Yujia Pan, Jinghui Wu, Shanshan Tie, Xuan Li, Li Cao, Yao Dong, Jianguo Zhu, Shuguang Fang, Ying Wu, Shaobin Gu

**Affiliations:** 1 College of Food and Bioengineering, Henan University of Science and Technology, Luoyang 471023, China; 2 Henan Engineering Research Center of Food Microbiology, Luoyang 471023, China; 3 Wecare Probiotics R&D Centers (WPC), Wecare Probiotics Co., Ltd., Suzhou 215200, China; 4 Henan Provincial Key University-Enterprise Joint R&D Center of Probiotics Scientific Evidence-Based Research and Industrial Application, Luoyang 471023, China

**Keywords:** *Weizmannia coagulans*, obesity, gut microbiota, inflammatory factors, untargeted metabolomics, lysophosphatidylcholines

## Abstract

**Highlights:**

**What are the main findings?**

- The *Weizmannia coagulans* BC99 strain improves inflammation in obese rats and corrects defects in gut immune function.
- BC99 remodels the gut microbiota and modulates key metabolites, including SCFAs, anti-inflammatory lysophosphatidylcholines, and conjugated bile acids.

**What are the implications of the main findings?**

- These findings establish a “microbiota–metabolite–host” axis as a key mechanism underlying the anti-obesity effects of *W. coagulans* BC99.
- These multi-omics-based findings highlight the potential of *W. coagulans* BC99 for use in obesity management.

**Abstract:**

**Background**: Obesity is closely related to dysbiosis. Probiotics may improve metabolism and alleviate inflammation by regulating microbial–host interaction. **Methods**: Obesity was induced in rats by feeding a high-fat diet, followed by gavage administration of varying doses of BC99 as an intervention. **Results**: BC99 significantly reduced body weight gain, improved lipid profiles, alleviated systemic inflammation, and enhanced gut barrier integrity. 16S rRNA sequencing revealed that BC99 increased the abundance of beneficial bacteria, including Bacillota, *Akkermansia,* and *Roseburia*. Untargeted metabolomics showed that BC99 upregulated anti-inflammatory lysophosphatidylcholines (LysoPCs) and modulated conjugated bile acids (GUDCA, GDCA), which were correlated with enriched bile salt hydrolase (BSH)-active bacteria (e.g., *Lachnoclostridium*). **Conclusions**: The results indicate that *W. coagulans* BC99 effectively reduces weight gain in rats made obese by a high-fat diet and improves metabolic disorders. These effects are associated with remodeling of the gut microbiota and modulation of key metabolites, supporting a potential ‘microbiota–metabolite–host’ axis in rats that warrants further causal validation.

## 1. Introduction

Obesity represents a critical and escalating global health challenge. Recent estimates indicate that over one billion individuals worldwide are affected, underscoring the severity of this epidemic [1]. In China, the prevalence of overweight and obesity has risen sharply, now impacting more than 50% of adults and nearly 20% of school-aged children [2]. This trend not only imposes a substantial burden of disease but also significantly compromises quality of life and socioeconomic development. Beyond its physical manifestations, obesity plays a central role in the pathogenesis of type 2 diabetes, several types of cancer, and nonalcoholic fatty liver disease. Its disruptive effects on systemic metabolic homeostasis are mediated by persistent, chronic low-grade inflammation and insulin resistance [3].

Emerging research has established that gut microbiota dysbiosis is a significant contributor to obesity development [4]. Characteristic features of the obese gut microbiome include reduced diversity, a higher Bacillota/Bacteroidota ratio, and a depletion of beneficial bacteria capable of producing short-chain fatty acids (SCFAs) [5]. Such dysbiosis exacerbates metabolic dysfunction through multiple mechanisms: it enhances dietary energy harvest [6], compromises intestinal barrier integrity, and promotes the translocation of endotoxins into circulation, leading to metabolic endotoxemia and systemic inflammation [7]. Furthermore, altered microbial metabolites, including SCFAs, can modulate gut–brain axis signaling and bile acid metabolism, thereby interfering with host energy balance and appetite regulation [8].

Defined as live microorganisms that confer health benefits when administered in sufficient quantities, probiotics have emerged as a promising strategy to remodel the gut microbiota and combat obesity [9]. Their beneficial actions include competitive exclusion of pathogens, restoration of gut barrier function, modulation of microbial community structure, and production of bioactive metabolites such as butyrate, which directly influences host metabolism and immune responses [10,11]. Substantial preclinical evidence supports the anti-obesity potential of various probiotic strains. For instance, *Lactobacillus plantarum* HAC01 reduced mesenteric fat accumulation and up-regulated genes involved in lipid oxidation [12]. *Lactobacillus reuteri* J1 prevented weight gain, ameliorated dyslipidemia, and optimized glucose homeostasis, with these benefits correlating with changes in gut microbiota and bile acid composition [13]. Similarly, *Lactobacillus plantarum* Y44 strengthened colonic tight junctions, down-regulated pro-inflammatory signaling, and favorably modulated the gut microbial profile in obese mice [14].

Among various probiotics, spore-forming bacteria such as *Weizmannia coagulans* (formerly *Bacillus coagulans*) offer distinct advantages. Due to its robust spore coat, *W. coagulans* exhibits high survival rates during gastrointestinal transit, enabling transient colonization—a trait that may reduce safety concerns associated with permanent gut residency. Importantly, this transient presence does not preclude sustained biological effects. During its limited colonization window, *W. coagulans* can modulate the gut ecosystem by creating favorable niches and engaging in cross-feeding interactions, thereby promoting the proliferation of beneficial indigenous bacteria such as *Akkermansia* and *Roseburia* [15,16]. These ecological shifts, in turn, enhance intestinal barrier function and metabolic homeostasis, effects that may persist even after the probiotic itself is cleared. For instance, specific strains like *Bacillus coagulans* T4 have been shown to attenuate obesity-induced white adipose tissue inflammation in murine models [17], highlighting the genus’s therapeutic potential. However, the precise mechanisms underlying these sustained benefits—particularly the integrated microbiota–metabolite–host interactions—remain incompletely understood and warrant further investigation.

*Weizmannia coagulans* possesses superior tolerance to heat, acid, and bile compared to commonly used lactic acid bacteria, which ensures its high viability during product storage and gastrointestinal transit. The strain under investigation, *W. coagulans* BC99, has shown promising probiotic attributes in preliminary assessments, including robust survival and adhesion capabilities. Nevertheless, a comprehensive evaluation of its efficacy against diet-induced obesity, coupled with a systems-level analysis to decipher its mode of action, has not been previously reported.

Therefore, the present study systematically investigated the therapeutic effects of *W. coagulans* BC99 on high-fat diet-induced obesity in rats. We employed a combination of 16S rRNA gene sequencing and untargeted serum metabolomics—with a particular emphasis on identifying microbial-derived metabolites and their host interactions—to test the hypothesis that BC99 alleviates obesity and associated metabolic disturbances in HFD-fed rats, potentially through remodeling gut microbiota composition and modulating key metabolites, while acknowledging that causality remains to be established. Our integrated multi-omics approach aims to provide a mechanistic framework for BC99’s anti-obesity effects, offering new insights into developing targeted probiotic interventions.

## 2. Materials and Methods

### 2.1. Experimental Animals and Grouping

Ninety specific pathogen-free (SPF) male Sprague–Dawley rats (4 weeks old, 180–200 g) were purchased from Spefford Biotechnology Co., Ltd. (Henan, China). The animal room was kept at a temperature of 22 ± 2 °C, a relative humidity of 45 ± 10%, and a 12 h light/12 h dark cycle. All experimental procedures received approval from the Laboratory Animal Ethics Committee of Henan University of Science and Technology and were performed in accordance with the ARRIVE Guidelines 2.0 [18].

### 2.2. Materials and Equipment

*Weizmannia coagulans* BC99 strain was obtained from Wecare Probiotics Co., Ltd. (Suzhou, China). Enzyme-linked immunosorbent assay (ELISA) kits for rat interleukin-6 (IL-6), interleukin-1β (IL-1β), tumor necrosis factor-α (TNF-α), interleukin-10 (IL-10), nitric oxide (NO), lipopolysaccharides (LPS), glucose, glucagon-like peptide-1 (GLP-1), glucagon-like peptide-2 (GLP-2), total cholesterol (TC), triglycerides (TG), low-density lipoprotein cholesterol (LDL-C), high-density lipoprotein cholesterol (HDL-C), alanine aminotransferase (ALT), and aspartate aminotransferase (AST) were purchased from Hepeng Biotechnology Co., Ltd. (Shanghai, China). ELISA kits for Zonula Occludens-1 (ZO-1) and Occludin were purchased from Uping Biotechnology Co., Ltd. (Hangzhou, China).

### 2.3. Intervention Plan

After a one-week acclimatization period, and based on the 3R principle for animal experimentation and power analysis, a sample size of 10 animals per group was determined, and the rats were subsequently randomly divided into nine groups [19]. Five groups were fed a high-fat diet (45% kcal from fat; purchased from Beijing Boaigang Biological Technology Co., Ltd.) (Beijing, China) to induce obesity: the model group (HFD), low-dose BC99 group (BC99_L), medium-dose BC99 group (BC99_M), high-dose BC99 group (BC99_H), and the orlistat positive control group (ORL). The remaining four groups received a standard normal diet (purchased from SPF Biotechnology Co., Ltd.) (Henan, China): the control group (CON), control + low-dose BC99 group (BC99_LC), control + medium-dose BC99 group (BC99_MC), and control + high-dose BC99 group (BC99_HC). Following eight weeks of dietary intervention, the successful generation of the obese phenotype was confirmed by a 20% increase in body weight in the HFD-fed groups compared to the normal-diet groups.

Subsequently, an eight-week oral intervention phase began. The dosages of *W. coagulans* BC99 (2 × 10^8^, 2 × 10^9^, and 2 × 10^10^ CFU/kg body weight for low-, medium-, and high-doses, respectively) were determined by converting the effective dose from a previous human clinical intervention trial of BC99 to the equivalent range for rats. Accordingly, rats in the BC99_L, BC99_M, and BC99_H groups (HFD-fed), as well as those in the BC99_LC, BC99_MC, and BC99_HC groups (normal diet-fed), received a daily gavage of BC99 at their respective designated doses. The ORL group received orlistat at 60 mg/kg body weight daily. The CON and HFD groups were administered an equal volume of 0.9% saline solution daily.

### 2.4. Histopathological Testing

Rat liver, adipose tissue, and intestinal samples were collected, washed with phosphate-buffered saline (PBS), and then immersed in 4% paraformaldehyde for 48 h. After fixation, the samples were processed through paraffin embedding and sectioning. Tissue sections were then stained with hematoxylin and eosin (H&E). Histopathological examination was performed using a light microscope (E100, Nikon Corporation, Tokyo, Japan) under brightfield illumination.

### 2.5. Determination of Inflammatory Factors

Serum was isolated from rat blood samples via centrifugation at 4 °C (4000× *g* for 15 min). The concentrations of IL-6, IL-1β, TNF-α, LPS, NO, and IL-10 in serum were measured using ELISA kits in strict accordance with the manufacturers’ protocols. Kits to measure these were acquired from Hepeng Biotechnology Co., Ltd. (Shanghai, China).

### 2.6. Glucose Metabolism-Related Indicators and Tolerance Tests

Following eight weeks of intervention, serum samples were harvested from rat blood via centrifugation at 4 °C (4000× *g* for 15 min). Glucose concentrations in serum were quantified utilizing a commercially available assay kit (Hepeng Biotechnology Co., Ltd., Shanghai, China), strictly adhering to the manufacturer’s guidelines. Fasting blood glucose levels were assessed following a 12–14 h overnight fast by sampling from the tail vein using a glucometer. Circulating levels of GLP-1 and GLP-2 were measured employing ELISA kits (Hepeng Biotechnology Co., Ltd., Shanghai, China), with all procedures executed in accordance with the supplier’s instructions.

An oral glucose tolerance test was conducted during the 7th week of the experimental period. Following an overnight fast, animals were administered a glucose load (2 g/kg body weight) via oral gavage. Glycemia was monitored in blood obtained from the tail vein immediately prior to administration (0 min), as well as at 30, 60, 90, and 120 min post-administration.

An insulin tolerance test was performed in the 8th week. After an overnight fasting period, the rats received an intraperitoneal injection of insulin (0.75 U/kg body weight). Blood glucose concentrations were recorded immediately prior to the injection (0 min) and at 15, 30, 60, 90, and 120 min thereafter.

### 2.7. Measurement of ZO-1 and Occludin Levels in Serum and Colon Tissue

Serum levels of ZO-1 and occludin were measured using ELISA kits, following the manufacturer’s guidelines. Blood samples were processed to obtain serum as detailed in Section 2.5. For colonic tissue, specimens were collected, washed with ice-cold PBS, and homogenized in PBS containing a protease inhibitor cocktail. The homogenate was then centrifuged at 12,000× *g* for 15 min at 4 °C, and the resultant supernatant was harvested for ELISA. All ELISA kits employed in this investigation were procured from Uping Biotechnology Co., Ltd. (Hangzhou, China). The concentrations of ZO-1 and occludin were derived from standard curves generated using the calibrators supplied with the kits.

### 2.8. 16S rRNA Sequencing Analysis

After the experiment, cecal contents were collected into sterile centrifuge tubes, quickly frozen in liquid nitrogen, and stored at −80 °C. DNA was isolated from the samples using the CTAB method. The purity and concentration of the extracted DNA were assessed by 1% agarose gel electrophoresis. The V3-V4 hypervariable region of the bacterial 16S rRNA gene was amplified using primer pair 341F and 805R. Sequencing libraries were prepared using the NEB Next^®^ Ultra DNA Library Prep Kit (New England Biolabs, Ipswich, MA, USA). Library quality and quantity were assessed using an Agilent 5400 system (Agilent Technologies, Santa Clara, CA, USA) and quantitative PCR. Qualified libraries were sequenced on the Illumina platform (Illumina, Inc., San Diego, CA, USA).

### 2.9. Untargeted Metabolomics Analysis by LC-MS

For untargeted metabolomic profiling, liquid chromatography–mass spectrometry (LC-MS) was conducted using an instrument from Microtech (Shenzhen, China). Tissue samples (100 mg), previously pulverized in liquid nitrogen were transferred into microcentrifuge tubes and extracted with 500 μL of 80% methanol in water. Following vortex mixing, the suspension was maintained on ice for 5 min, followed by centrifugation. An appropriate volume of the resulting supernatant was subsequently adjusted with LC-MS-grade water to reduce the methanol proportion to 53%. This diluted solution was then subjected to a second centrifugation step under identical conditions (15,000× *g*, 4 °C, 20 min), and the cleared supernatant was retained for instrumental analysis [20]. Separation was achieved on a Thermo Fisher (Bremen, Germany) column integrated with a Vanuquish UHPLC system. The mass spectrometric analysis was carried out using a Q Exactive™ HF or Q Exactive™ HF-X mass spectrometer (Thermo Fisher, Bremen, Germany). The chromatographic mobile phases comprised 1% formic acid in water (A) and methanol (B). MS/MS data were acquired in data-dependent acquisition mode. Raw spectral data were processed within the Compound Discoverer 3.1 software environment (Thermo Fisher), which facilitated peak alignment, feature detection, and metabolite identification. Putative metabolite annotations were established by referencing the KEGG (https://www.genome.jp/kegg/pathway.html, accessed on 8 July 2025), HMDB (https://hmdb.ca/metabolites, accessed on 8 July 2025), and LIPIDMaps (http://www.lipidmaps.org/, accessed on 8 July 2025) databases.

To ensure data reliability and reproducibility, a series of quality control (QC) measures were implemented. Pooled QC samples were prepared by mixing equal aliquots of all samples and injected periodically throughout the analytical run to monitor instrument stability and signal drift. Principal component analysis (PCA) of QC and test samples showed tight clustering of QCs, indicating good system stability. For test samples, those falling outside the 95% confidence interval in the PCA score plot were considered potential outliers; any sample with a PC1 value exceeding the mean ± 3 standard deviations was excluded to avoid interference with subsequent analyses. Signal drift and batch effects were corrected using the QC-RFSC algorithm (R package statTarget 1.16.1), which markedly improved QC clustering in PCA. Data preprocessing involved three steps: (i) within-sample normalization by dividing each metabolite’s peak area by the sample median to remove between-sample total ion intensity differences; (ii) natural log transformation to approximate a normal distribution; and (iii) Pareto scaling to render metabolites of different magnitudes comparable in multivariate analysis. Metabolite identification confidence was assigned according to Metabolomics Standards Initiative levels: Level 1 confirmed by authentic standards (highlighted in red in the exported Excel file), Level 2 putatively annotated based on MS/MS spectral matching with NIST, Wiley Registry, HMDB, and LIPID MAPS databases, and Level 3 putatively characterized based on accurate mass and chromatographic behavior. In subsequent differential analyses of metabolites and microbiota, statistical significance was set at q < 0.05 after Benjamini–Hochberg FDR correction.

For multivariate statistical analysis, the preprocessed data were subjected to OPLS-DA using SIMCA-P software (version 14.1, Umetrics, Sweden). Model validity was assessed by 7-fold cross-validation and permutation testing (200 permutations).

### 2.10. Determination of Rat Short-Chain Fatty Acids

SCFAs in the feces of rats were determined using a gas chromatograph and a JN-5MS column. The experimental parameters were as follows: the injector temperature was maintained at 250 °C, and the column oven temperature program was: initially held at 40 °C for 1 min, then increased to 60 °C at 8 °C/min and held for 1 min, then increased to 70 °C at 10 °C/min and held for 1 min, and finally raised to 220 °C at 20 °C/min and held for 10 min. The injection volume was 1 µL, and the column flow rate was 1.5 mL/min [21].

### 2.11. Statistical Analysis

All values are presented as mean ± standard error of the mean (SEM). Statistical evaluations were carried out utilizing GraphPad Prism 8 and SPSS 22.0 software packages. The assumption of normality was verified using the Shapiro–Wilk test. In cases involving comparisons across multiple groups, a one-way analysis of variance (ANOVA) was employed, coupled with Dunnett’s post hoc test to facilitate comparisons specifically against the HFD group; this approach inherently controls the family-wise error rate for multiple testing. For longitudinal datasets, including body weight trajectories, OGTT, and ITT, a two-way repeated measures ANOVA was conducted, incorporating Bonferroni’s post hoc correction to adjust significance thresholds for multiple comparisons. When assumptions of normality or homogeneity of variances were violated, the Kruskal–Wallis test served as the nonparametric alternative, with subsequent application of Dunn’s post hoc test, which includes correction for multiple comparisons. Associations between variables were evaluated using Spearman’s rank correlation coefficient. For all statistical assessments, a *p*-value of less than 0.05 was regarded as indicating statistical significance. Significance levels in figures are denoted as follows: * *p* < 0.05, ** *p* < 0.01, and *** *p* < 0.001.

## 3. Results

### 3.1. Effects of BC99 on Phenotypic Characteristics and Adipose Tissue Morphology in HFD-Induced Obese Rats

**Figure 1:**
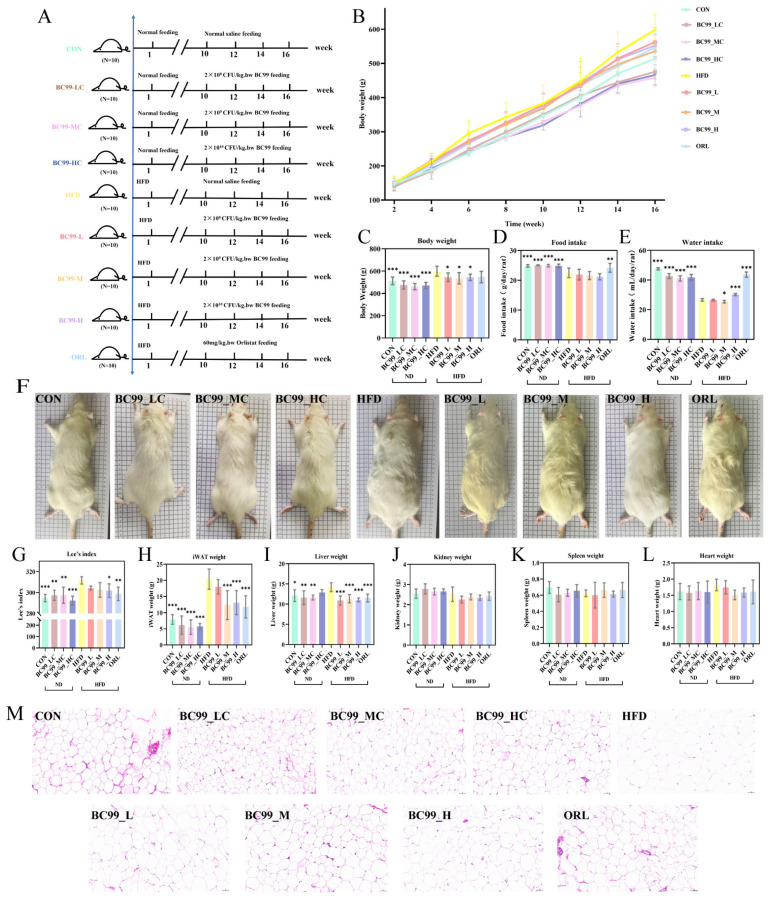
Effects of BC99 on body weight, adipose tissue, and organ weights in HFD-fed rats. (**A**) Experimental timeline. (**B**) Body weight growth curves over 16 weeks. (**C**) Final body weight. (**D**) Food intake. (**E**) Water intake. (**F**) Representative phenotypic images. (**G**) Lee’s index. (**H**) iWAT weight. (**I**) Liver weight. (**J**–**L**) Kidney, spleen, and heart weights. (**M**) H&E-stained iWAT sections (scale bar = 50 μm). Data are mean ± SEM. * *p* < 0.05, ** *p* < 0.01, *** *p* < 0.001 vs. HFD group.

After eight weeks of HFD feeding, body weight in the HFD group increased by 20% compared to the control group (*p* < 0.01), confirming successful obesity induction. Following eight weeks of BC99 intervention, significant body weight differences were observed between the HFD group and all treatment groups (CON, BC99_L, BC99_M, BC99_H) (*p* < 0.05) (Figure 1B,C), with corresponding phenotypic changes (Figure 1F).

Under ad libitum feeding conditions, total energy intake in the HFD group exceeded that in the CON group due to the higher energy density of the high-fat diet. Consequently, although the CON group consumed a greater mass of food, its total energy intake was lower, sufficient only to maintain normal body weight [22]. The results showed that rats treated with the drug and those on a normal diet exhibited significantly increased food intake (*p* < 0.01) (Figure 1D).

In the obesity model, the CON group exhibited higher water intake than the HFD group. This difference can be attributed to the lower demand for exogenous water in HFD-fed rats, which produce more endogenous metabolic water through fat oxidation, alongside other factors such as the physical properties of the high-fat diet and altered gut microbiota. Compared with the HFD group, the water intake of CON, BC99_LC, BC99_MC, BC99_HC, BC99_M, BC99_H, and ORL groups increased significantly (*p* < 0.05) (Figure 1E) [23,24].

The Lee’s index, a body-weight-to-length ratio used to assess obesity while correcting for body size, was significantly reduced in the high-dose intervention group (*p* < 0.05) (Figure 1G). Adipose tissue mass was also markedly reduced: with the exception of the BC99_L group, the weight of inguinal white adipose tissue (iWAT) in all BC99 treatment groups was significantly lower than that in the HFD group (*p* < 0.001) (Figure 1H). Similarly, the HFD-induced increase in liver weight was significantly reversed in all BC99 and ORL groups, with liver mass returning to levels close to those of the CON group (*p* < 0.05) (Figure 1I). In contrast, no significant differences in kidney, spleen, or heart weights were observed among the groups (Figure 1J–L). Histological examination of iWAT (H&E staining) revealed adipocyte hypertrophy in the HFD group, accompanied by extensive macrophage infiltration. The mean adipocyte cross-sectional area in the HFD group was significantly larger than that in the CON group. In contrast, BC99 intervention reduced adipocyte size in a dose-dependent manner (Figure 1M).

### 3.2. BC99 Ameliorates Metabolic and Inflammatory Markers and Hepatic Pathological Injury

**Figure 2:**
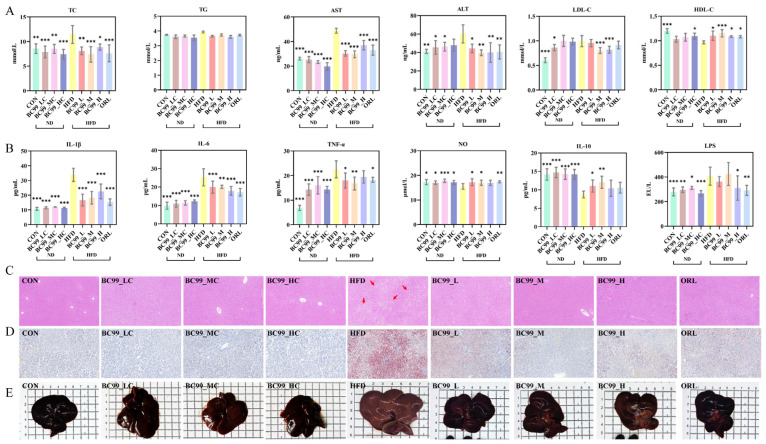
Effects of BC99 on serum parameters, inflammatory cytokines, and liver histology in HFD-fed rats. (**A**) Serum TC, TG, AST, ALT, LDL-C and HDL-C. (**B**) Serum IL-1β, IL-6, TNF-α, NO, IL-10, and LPS. (**C**) Liver H&E staining, scale bar = 100 μm (red arrows: vacuoles from lipid droplet dissolution). (**D**) Liver Oil Red O staining, Scale bar = 100 μm. (**E**) Gross liver morphology. Data are mean ± SEM. * *p* < 0.05, ** *p* < 0.01, *** *p* < 0.001 vs. HFD group.

Compared with the CON group, the HFD group showed significantly higher serum TC and LDL-C levels after 8 weeks of feeding, while HDL-C levels were lower. Intervention with the BC99_L, BC99_M, BC99_H or ORL group significantly ameliorated this dyslipidemia (*p* < 0.05), although TG levels were not significantly altered (Figure 2A).

HFD feeding for 8 weeks significantly elevated serum levels of LPS, TNF-α, IL-6, and IL-1β, while markedly reducing the level of the anti-inflammatory cytokine IL-10 (*p* < 0.05). BC99 treatment at all doses significantly reversed these HFD-induced alterations (*p* < 0.05) (Figure 2B).

HFD feeding induced significant hepatic injury, as evidenced by serum AST and ALT levels that were significantly elevated above normal levels (Figure 2A). Gross examination revealed that livers from HFD-fed rats were pale and enlarged. HE staining revealed that obesity modeling induced hepatocyte steatosis and inflammatory cell infiltration, while Oil Red O staining further confirmed significant lipid accumulation in the liver. BC99 intervention, particularly at the medium dose, significantly attenuated these pathological changes, as reflected by reduced liver weight, improved serum transaminase levels, and ameliorated hepatic steatosis and lipid accumulation (*p* < 0.05) (Figure 2C–E).

### 3.3. Effects of BC99 on Glucose Metabolism in Rats

**Figure 3:**
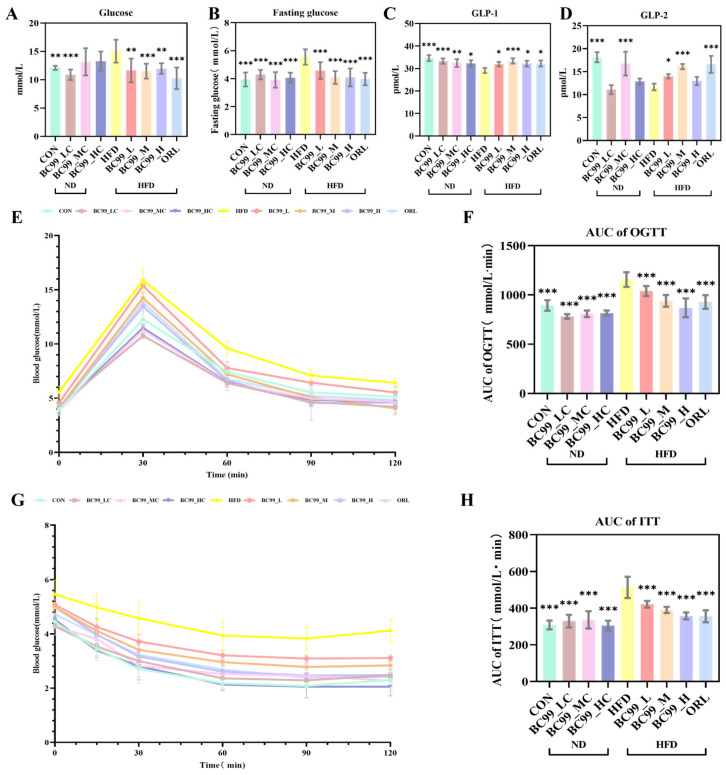
Effects of BC99 on glucose metabolism and insulin sensitivity in HFD-fed SD rats. (**A**) Non-fasting serum glucose levels. (**B**) FBG levels. (**C**) GLP-1 levels. (**D**) GLP-2 levels. (**E**) OGTT. (**F**) AUC of OGTT. (**G**) ITT. (**H**) AUC of ITT. Data are mean ± SEM. * *p* < 0.05, ** *p* < 0.01, *** *p* < 0.001 vs. HFD group.

After 8 weeks of intervention, fasting blood glucose (FBG) levels were significantly improved in all BC99-treated groups (*p* < 0.001) (Figure 3B). Non-fasting serum glucose levels showed a similar downward trend (*p* < 0.01) (Figure 3A).

An OGTT was performed at week 7. Throughout the 120 min test, blood glucose levels in the HFD group remained higher than those in the other eight groups. In contrast, the area under the curve (AUC) for glucose was significantly reduced in all BC99-treated groups (*p* < 0.001) (Figure 3E,F). Following BC99 intervention, serum GLP-1 levels were significantly restored in all treated groups (*p* < 0.05) (Figure 3C). Furthermore, the BC99_L and BC99_M groups showed significant improvement in serum GLP-2 levels (*p* < 0.05) (Figure 3D).

An ITT was conducted at week 8. At all measured time points, blood glucose levels in the HFD group were higher than those in the other eight groups. At 30 min post-injection, all BC99-treated groups showed significantly lower blood glucose levels (Figure 3G). Consistently, the AUC of glucose during the ITT was significantly lower in the BC99-treated groups than in the HFD group (*p* < 0.001), demonstrating that all three doses of BC99 effectively attenuated the HFD-induced elevation in blood glucose levels (*p* < 0.001) (Figure 3H).

### 3.4. Improvement Effect of BC99 on Intestinal Injury in Obese Rats

**Figure 4:**
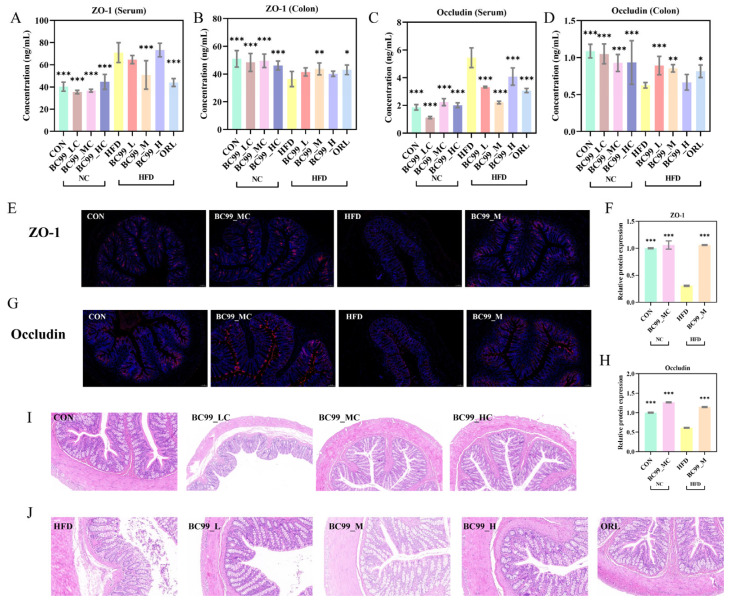
Effects of BC99 on intestinal barrier integrity in HFD-fed SD rats. (**A**) Serum ZO-1 levels. (**B**) Colonic ZO-1 protein expression levels. (**C**) Serum occludin levels. (**D**) Colonic occludin protein expression levels. (**E**) Representative immunofluorescence staining of ZO-1 in colon tissue (scale bar = 100 μm, Blue: DAPI, Red: target protein). (**F**) Relative fluorescence intensity of ZO-1. (**G**) Representative immunofluorescence staining of occludin in colon tissue (scale bar = 100 μm, Blue: DAPI, Red: target protein). (**H**) Relative fluorescence intensity of occludin. (**I**,**J**) Representative H&E-stained sections of colon tissue (scale bar = 100 μm). Data are mean ± SEM. * *p* < 0.05, ** *p* < 0.01, *** *p* < 0.001 vs. HFD group by one-way ANOVA with Dunnett’s post hoc test.

The BC99-M group significantly reduced the obesity-induced elevation in serum ZO-1 levels. (*p* < 0.001) (Figure 4A). All BC99-treated groups significantly reduced serum occludin levels (*p* < 0.001) (Figure 4C). Furthermore, all BC99-treated groups significantly increased the protein expression levels of ZO-1 and occludin in colon tissue (*p* < 0.05; Figure 4B,D).

Immunofluorescence staining showed that ZO-1 and occludin were continuously and clearly distributed along the apical side of colonic epithelial cells in the CON group. The HFD group exhibited a discontinuous, fragmented, and markedly weaker fluorescent signal. BC99 intervention at the medium dose (BC99_M) restored the continuity and intensity of the fluorescent signal to a pattern comparable to that of the CON group (Figure 4E,G). Quantitative analysis confirmed that the relative fluorescence intensities of ZO-1 and occludin were significantly higher in the BC99_M group than in the HFD group (*p* < 0.001) (Figure 4F,H).

H&E staining of colonic tissue revealed intact mucosal architecture, orderly glandular arrangement, and no evident inflammatory infiltration in the CON group. The HFD group showed pronounced inflammatory cell aggregation, mucosal thickening or disruption, and disorganized glandular structures. BC99 intervention markedly ameliorated these histopathological alterations, with tissue morphology restored to a near-normal appearance (Figure 4I,J).

### 3.5. BC99 Can Improve the Composition and Abundance of Intestinal Microbiota in HFD-Induced Obese Rats

**Figure 5:**
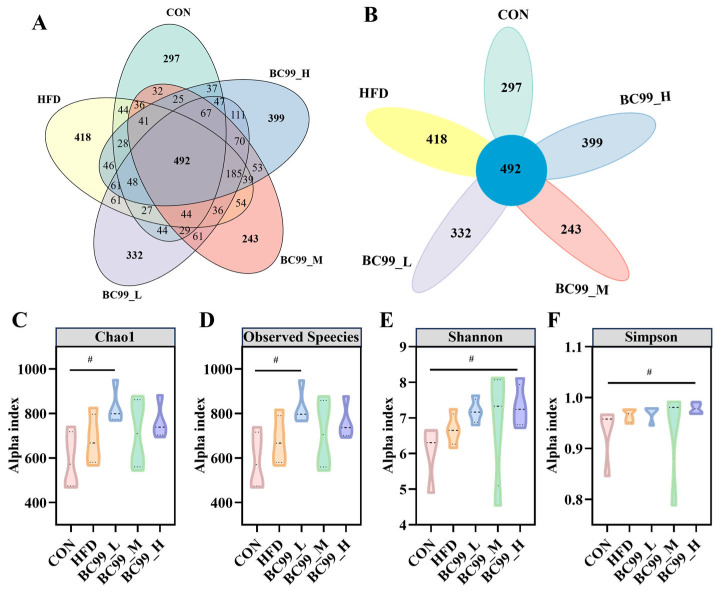
Effects of BC99 on gut microbiota diversity and composition in HFD-induced obese rats. (**A**,**B**) Venn diagrams illustrating shared and unique OTUs across groups. (**C**) Chao1 index. (**D**) Observed species index. (**E**) Shannon index. (**F**) Simpson index (violin plot). Data are mean ± SEM. ^#^ *p* < 0.05 vs. CON group.

Following an 8-week high-fat diet intervention, a total of 1689 operational taxonomic units (OTUs) were detected across all samples. Of these, 492 OTUs were shared among all groups. The BC99 low-, medium-, and high-dose groups had 332, 243, and 399 unique OTUs, respectively, while the HFD group had 418 unique OTUs. The CON group shared 37 OTUs with the high-dose BC99 group and 32 OTUs with the medium-dose group; in contrast, the CON group shared only 44 OTUs with the HFD group (Figure 5A,B).

Alpha diversity analysis revealed that the observed species and Chao1 indices were significantly higher in the BC99_L group than in the CON group (*p* < 0.05) (Figure 5C,D). The Shannon and Simpson indices in the BC99 high-dose group were also significantly higher than those in the CON group (*p* < 0.05) (Figure 5E,F). These results indicate that BC99 intervention, at specific doses, enhanced the diversity and richness of the gut microbiota in HFD-fed rats.

**Figure 6:**
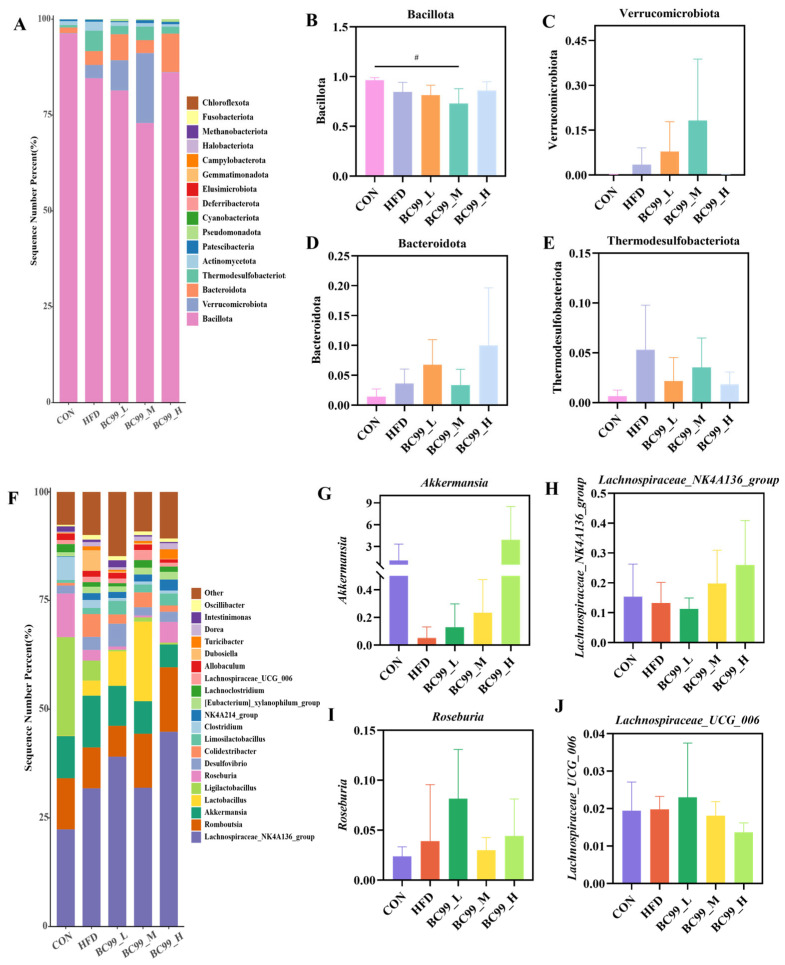
BC99 can improve the composition and abundance of intestinal microbiota in HFD-induced obese rats. (**A**) Taxonomic analysis of the microbiota in cecal content samples at the phylum level. (**B**–**E**) Phylum-level strains. (**F**) Taxonomic analysis of the microbiota in cecal content samples at the genus level. (**G**–**J**) Genus-level strains. ^#^ *p* < 0.05 vs. the CON group.

To further explore the impact of BC99 on the gut microbiota, we analyzed changes in bacterial composition at the phylum and genus levels using 16S rRNA gene sequencing data. At the phylum level, Bacillota and Verrucomicrobiota together accounted for more than 95% of the microbiome across all groups. Compared with the CON group, the average relative abundance of Bacillota in the BC99_M group was significantly reduced (*p* < 0.05) (Figure 6B). Following BC99 intervention, Verrucomicrobiota and Bacteroidota showed increasing trends to varying degrees relative to the HFD group, while Thermodesulfobacteriota exhibited a decreasing trend (Figure 6C–E). At the genus level, BC99 intervention enriched several beneficial bacteria. Compared with the HFD group, the relative abundances of *Akkermansia*, *Roseburia*, *Lachnospiraceae_NK4A136_group*, and *Lachnospiraceae_UCG-006* in BC99-treated groups showed varying degrees of increase (Figure 6G–J). These results suggest that BC99 intervention may partially ameliorate HFD-induced gut microbiota dysbiosis.

**Figure 7:**
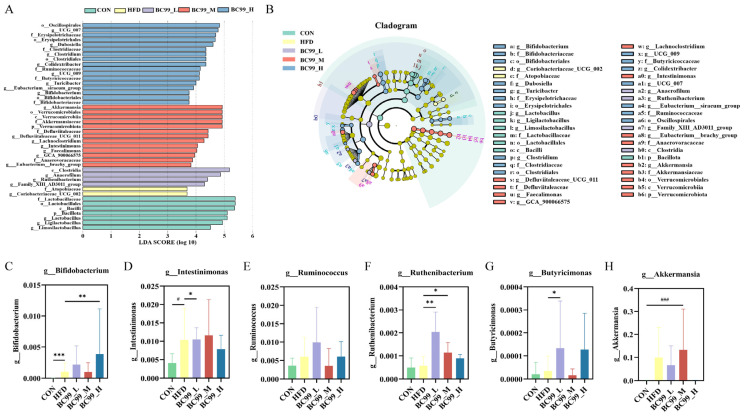
LEfSe analysis identifies differential gut microbial taxa among groups. (**A**) Histogram of linear discriminant analysis (LDA) scores (threshold > 2.0) for taxa with significant differences between groups. (**B**) Cladogram showing the phylogenetic distribution of differentially abundant taxa. (**C**–**H**) Relative abundances of six obesity-related genera selected from LEfSe results: (**C**) g_Bifidobacterium, (**D**) g_Intestinimonas, (**E**) g_Ruminococcaceae, (**F**) g_Ruthenibacterium, (**G**) g_Butyricimonas, (**H**) g_Akkermansia. Data are presented as mean ± SEM. ^#^ *p* < 0.05, ^###^ *p* < 0.001 vs. CON group; * *p* < 0.05, ** *p* < 0.01, *** *p* < 0.001 vs. HFD group.

Next, LEfSe analysis was performed to identify differentially abundant gut microbial taxa among the groups. Based on the LEfSe results, six obesity-related genera were selected for further analysis (Figure 7C–H). After eight weeks of intervention, the relative abundance of *Bifidobacterium* in the BC99_H group was significantly higher than that in the HFD group (*p* < 0.01) (Figure 7C). The abundance of *Intestinimonas* in the BC99_L group was significantly higher than that in the HFD group (*p* < 0.05) (Figure 7D). As shown in Figure 7E, although no significant difference was observed in the abundance of *Ruminococcaceae* between the BC99_L or BC99_H groups and the HFD group, an increasing trend was noted. The levels of *Ruthenibacterium* in the BC99_L and BC99_M groups were significantly higher than those in the HFD group (*p* < 0.05) (Figure 7F). The abundance of *Butyricimonas* in the BC99_L group was significantly higher than that in the HFD group (*p* < 0.05) (Figure 7G). Additionally, after eight weeks of intervention, the abundance of *Akkermansia* in the BC99_H group was significantly higher than that in the CON group (*p* < 0.001) (Figure 7H). Collectively, these results indicate that BC99 reshapes the gut microbiota of HFD-fed rats.

**Figure 8:**
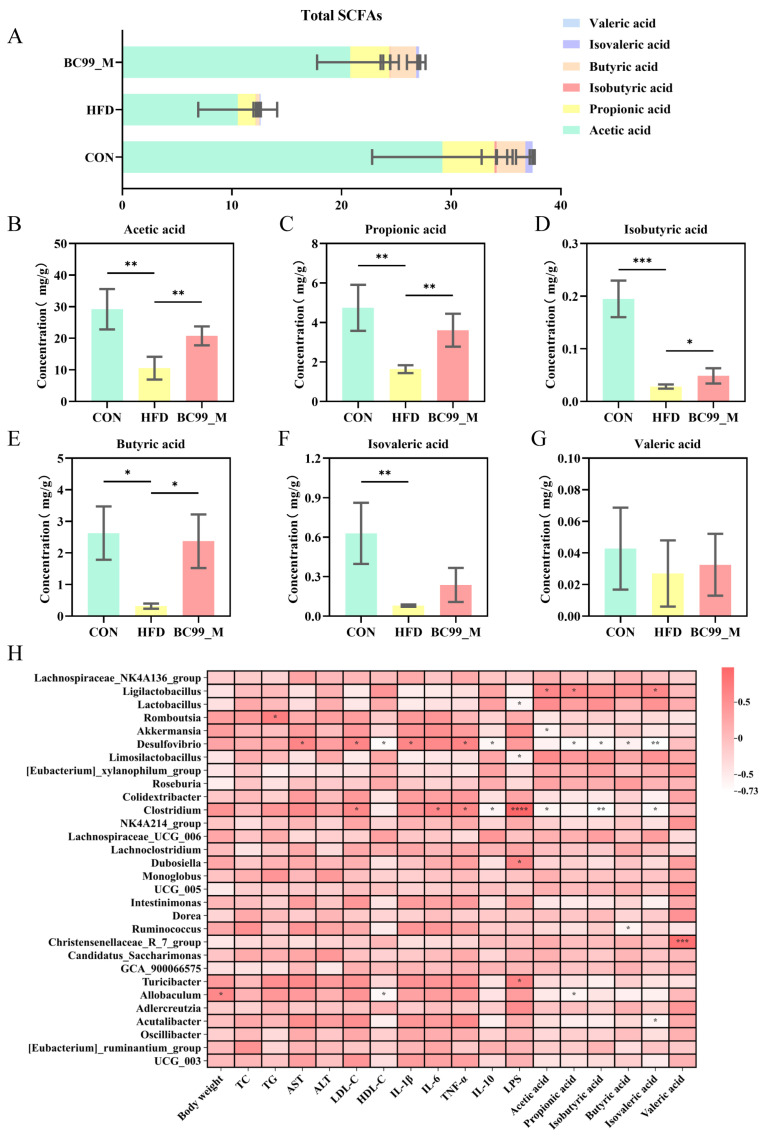
Effects of BC99 on fecal SCFA profiles and their correlations with biochemical parameters, inflammatory factors, and gut microbiota. (**A**) Total SCFAs and compositional distribution in each group (stacked bar chart). (**B**) Acetic acid levels. (**C**) Propionic acid levels. (**D**) Isobutyric acid levels. (**E**) Butyric acid levels. (**F**) Isovaleric acid levels. (**G**) Valeric acid levels. (**H**) Spearman correlation heatmap showing relationships among biochemical parameters, inflammatory factors, SCFAs, and genus-level gut microbiota. Data are mean ± SEM. * *p* < 0.05, ** *p* < 0.01, *** *p* < 0.001, **** *p* < 0.0001 vs. HFD group.

SCFAs serve as key mediators linking gut immunity and the microbiota. Studies have shown that obesity leads to a reduction in total fecal SCFA levels. Interestingly, after an eight-week intervention, the BC99_M group showed a significant increase in acetic acid (*p* < 0.01) (Figure 8B), propionic acid (*p* < 0.01) (Figure 8C), isobutyric acid (*p* < 0.05) (Figure 8D), and butyric acid (*p* < 0.01) (Figure 8E) compared with the HFD group. Although isovaleric acid and valeric acid did not show significant differences, they exhibited an upward trend (Figure 8F,G).

To elucidate the role of BC99 in alleviating obesity through modulation of the gut microbiota, this study employed Spearman correlation analysis to investigate the relationships among obesity-related biochemical parameters, gut microbiota, and inflammatory factors. As shown in Figure 8, both positive and negative correlations were observed among several biochemical parameters, bacterial genera, and inflammatory factors. Overall, body weight was positively correlated with *Allobaculum* and negatively correlated with *UCG_005*. TG was significantly positively correlated with *Lactobacillus* and *Limosilactobacillus*, while showing significant negative correlations with *Roseburia*, *UCG_005*, *Oscillibacter*, *Colidextribacter*, *Acutalibacter*, and *UCG_003*. TNF-α was positively correlated with *Desulfovibrio*, *Clostridium*, and *Turicibacter*. LPS was positively correlated with *Akkermansia*. Based on these results, these genera and inflammatory factors may serve as potential biomarkers for the alleviation of obesity. In conclusion, the anti-obesity effect of BC99 is associated with the modulation of the gut microbiota and inflammatory factors.

### 3.6. BC99 Improves the Serum Metabolic Profile of HFD-Induced Obese Rats

**Figure 9:**
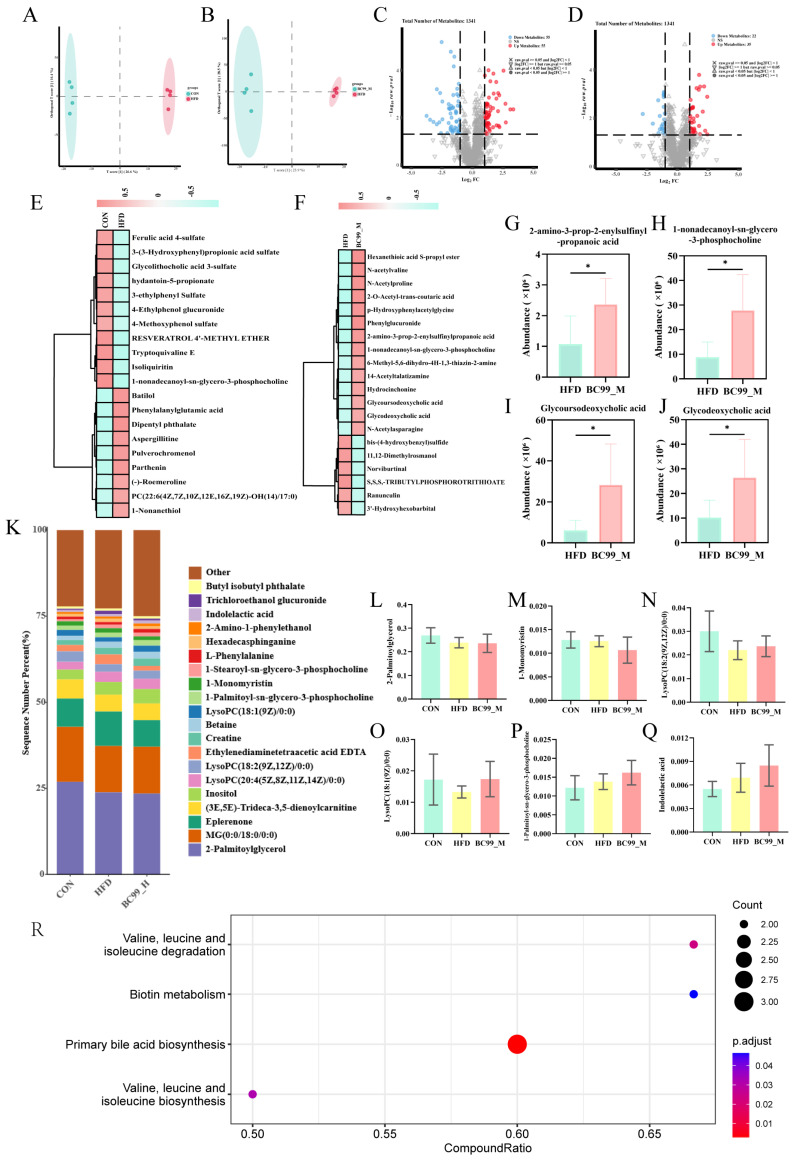
Effects of BC99 on serum metabolic profiles in HFD-induced obese rats. (**A**,**B**) OPLS-DA score plots showing separation between CON and HFD (**A**) and between HFD and BC99_M (**B**). (**C**,**D**) Volcano plots of differential metabolites (VIP > 1, *p* < 0.05) for CON vs. HFD (**C**) and HFD vs. BC99_M (**D**) Dashed lines indicate the significance threshold (q=0.05) and the fold change threshold ($|{log}_{2}\mathrm{FC}|=1$). (**E**,**F**) Hierarchical clustering heatmaps of differential metabolites. (**G**–**J**) Relative abundance of selected metabolites: (**G**) 2-amino-3-prop-2-enylsulfinylpropanoic acid, (**H**) 1-nonadecanoyl-sn-glycero-3-phosphocholine, (**I**) GUDCA, (**J**) GDCA. (**K**) Percentage stacked bar chart of key lipid metabolites. (**L**–**Q**) Relative abundance of individual metabolites from (**K**): (**L**) 2-palmitoylglycerol, (**M**) 1-monomyristin, (**N**) 1-stearoyl-sn-glycero-3-phosphocholine, (**O**) LysoPC(20:4), (**P**) LysoPC(18:1), (**Q**) indolelactic acid. (**R**) KEGG pathway enrichment analysis of altered metabolites. Data are mean ± SEM. * *p* < 0.05 vs. HFD.

To investigate the potential mechanisms underlying the anti-obesity effect of BC99, untargeted metabolomic analysis was performed on serum samples. The BC99_M group, which exhibited the most pronounced phenotypic improvement, was selected as the representative intervention group for comparison with the CON and HFD groups. OPLS-DA revealed distinct metabolic profiles between the CON and HFD groups, as well as between the HFD and BC99_M groups, indicating that BC99 intervention significantly altered the serum metabolome of HFD-fed rats (Figure 9A,B). We further performed an integrated OPLS-DA analysis including the CON, HFD, and BC99_M groups, and the score plot showed a clear separation among the three groups. Permutation testing confirmed that the model had no overfitting and exhibited good predictive ability (Figure S1).

Differential metabolites were identified based on VIP > 1 and *p* < 0.05. Compared with the CON group, 110 metabolites were differentially abundant in the HFD group, of which 55 were upregulated and 55 downregulated (Figure 9C). Compared with the HFD group, 57 metabolites were differentially abundant in the BC99_M group, including 22 upregulated and 35 downregulated metabolites (Figure 9D). Hierarchical clustering analysis of these differential metabolites is shown in Figure 9E,F.

Among the metabolites altered by BC99 intervention, several were selected for individual presentation based on their biological relevance (Figure 9G–J). Levels of 2-amino-3-prop-2-enylsulfinylpropanoic acid, a metabolite derived from organosulfur compounds, were increased in the BC99_M group compared with the HFD group (Figure 9G). ysophosphatidylcholine 1-nonadecanoyl-sn-glycero-3-phosphocholine was also elevated following BC99 treatment (Figure 9H). Additionally, the conjugated secondary bile acids glycoursodeoxycholic acid (GUDCA) and glycodeoxycholic acid (GDCA) were modulated by BC99 intervention (Figure 9I,J).

Grouped percentage stacked histograms further illustrated the relative abundance changes in key lipid metabolites (Figure 9K). Compared with the HFD group, BC99 intervention reduced the levels of 2-palmitoylglycerol, 1-monomyristin, and 1-stearoyl-sn-glycero-3-phosphocholine (Figure 9L–N). Furthermore, BC99 treatment decreased the levels of LysoPC(20:4) and LysoPC(18:1) (Figure 9O,P), while increasing the level of indolelactic acid (Figure 9Q). KEGG pathway enrichment analysis revealed that four pathways were significantly enriched following BC99 intervention: valine, leucine and isoleucine biosynthesis; primary bile acid biosynthesis; biotin metabolism; and valine, leucine and isoleucine degradation (impact value > 0.1, *p* < 0.05) (Figure 9R).

### 3.7. Correlation Analysis

**Figure 10:**
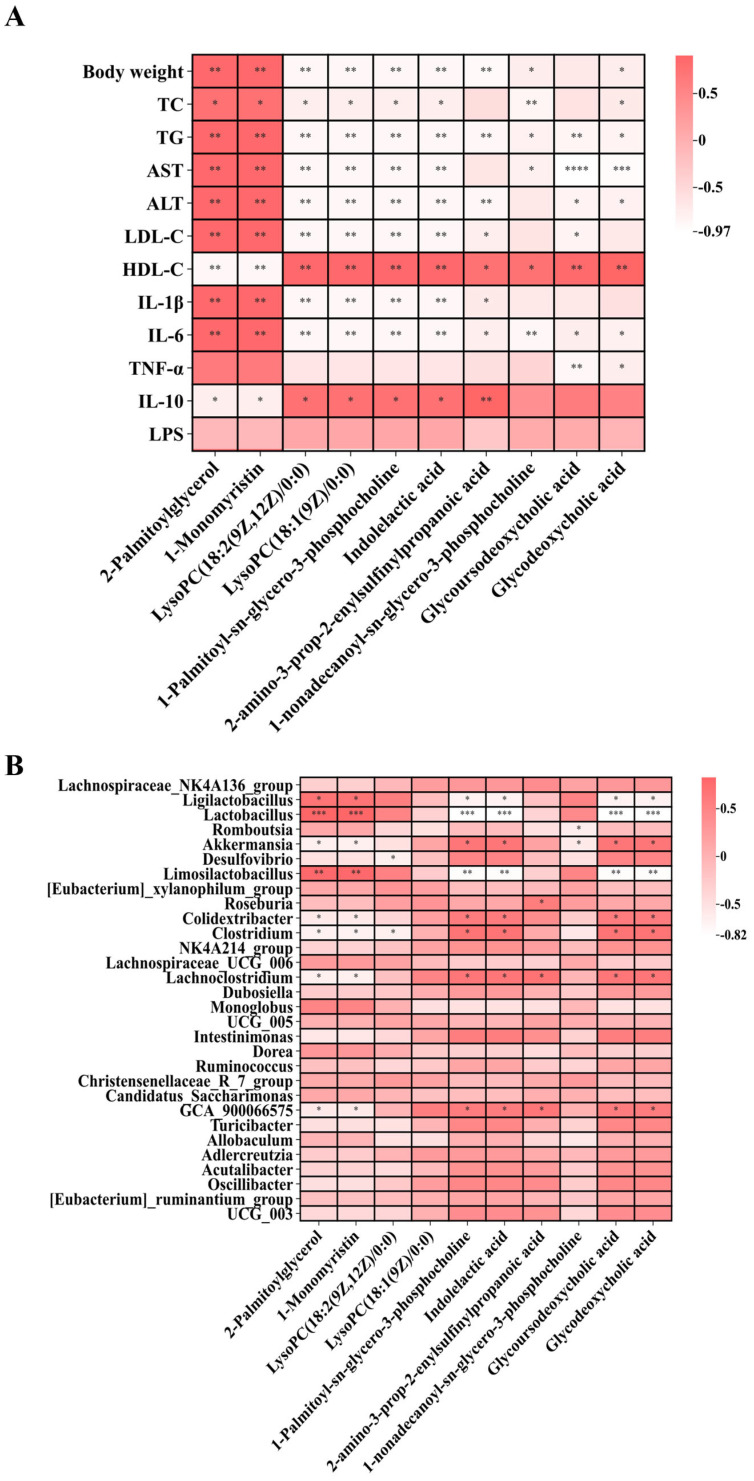
Correlation heatmaps of differential metabolites following BC99 intervention. (**A**) Correlation between differential metabolites and body weight, biochemical indices, inflammatory factors, and oxidative stress. (**B**) Correlation between differential metabolites and gut microbiota at the genus level. Correlation coefficients were calculated using Spearman’s rank correlation. * *p* < 0.05, ** *p* < 0.01, *** *p* < 0.001, **** *p* < 0.0001.

Subsequently, Spearman correlation analysis was performed to assess the associations between these differential metabolites and biochemical indicators or inflammatory factors (Figure 10A). LysoPC(18:2), LysoPC(18:1), 1-palmitoyl-sn-glycero-3-phosphocholine, indolelactic acid, 2-amino-3-prop-2-enylsulfinylpropanoic acid, glycoursodeoxycholic acid, and glycodeoxycholic acid were positively correlated with anti-inflammatory factors, while being negatively correlated with body weight and pro-inflammatory factors. Studies have shown that LysoPC(18:2) and LysoPC(18:1) exhibit potent anti-inflammatory properties [25]. LPS initiates downstream NF-κB and MAPK signaling pathways by binding to its receptor complex, leading to the release of pro-inflammatory factors [26]. Elevated levels of LysoPCs following BC99 intervention may contribute to repairing the gut barrier disrupted by the high-fat diet, thereby reducing LPS translocation from the gut into the bloodstream. This aligns with the observed inverse correlation between LysoPC levels and both circulating LPS and systemic inflammation [27]. Correlation analysis between differential metabolites and gut microbiota further revealed that glycoursodeoxycholic acid (GUDCA) and glycodeoxycholic acid (GDCA) were significantly positively correlated with *Lachnoclostridium* and *Akkermansia*. Some strains of *Lachnoclostridium* have been reported as important producers of bile salt hydrolase (BSH) in the gut [28]. Therefore, we speculate that BC99 supplementation increases the abundance of *Lachnoclostridium*, and the BSH produced by this genus may hydrolyze the amide bond of GUDCA, removing glycine to generate free ursodeoxycholic acid. Similarly, BSH may hydrolyze GDCA to produce free deoxycholic acid. This process not only alters the physicochemical properties of bile acids but also modulates their ability to activate bile acid receptors such as the farnesoid X receptor (FXR) and the G protein-coupled bile acid receptor (TGR5), thereby influencing host metabolic health [29]. These findings suggest that BC99-induced alterations in these potential biomarkers may contribute to ameliorating metabolic disorders and reducing inflammation in obese rats (Figure 10B).

## 4. Discussion

The global obesity epidemic is closely linked to gut microbiota dysbiosis, which contributes to metabolic inflammation and insulin resistance. Accumulating evidence has established that the gut microbiota forges a critical link between environmental factors and host metabolism by producing metabolites such as SCFAs, bile acids, and lipopolysaccharides, thereby regulating energy balance and immune responses [5,30]. Dysbiosis-induced intestinal barrier dysfunction and metabolic endotoxemia are recognized as core pathological features of obesity [31]. In this study, we observed that *W. coagulans* BC99 supplementation improved multiple obesity-related phenotypes in HFD-fed rats. By integrating 16S rRNA sequencing with untargeted metabolomics, we explored potential associations between gut microbiota shifts, metabolite changes, and host phenotypic improvements, providing a multi-level perspective on the potential involvement of a ‘gut microbiota–metabolite–host’ axis. It should be noted that the current evidence is correlative, and causality remains to be experimentally validated [32]. Interestingly, the medium dose (2 × 10^9^ CFU/kg) consistently outperformed the high dose (2 × 10^10^ CFU/kg) across multiple endpoints. This non-linear dose–response relationship is well documented in probiotic research [33], where effects may plateau or decline beyond an optimal threshold due to mechanisms such as saturation of intestinal adhesion sites or induction of immune tolerance [34]. Notably, the effective dose in a recent BC99 human trial (5 × 10^9^ CFU/day) corresponds to the medium-dose range in this study after allometric scaling, supporting its translational relevance [35]. These findings highlight the importance of dose optimization in future applications of BC99.

The gut microbiota is integral to intestinal barrier function and host metabolism. Modulating the microbiome has emerged as a promising strategy for combating obesity. In this study, BC99 intervention significantly altered the gut microbiota composition of HFD-fed rats, reducing the Bacillota/Bacteroidota ratio at the phylum level and enriching beneficial genera such as *Akkermansia*, *Roseburia*, and *Lachnospiraceae_NK4A136_group* at the genus level [36]. These microbial shifts were accompanied by increased fecal levels of acetic, propionic, butyric, and isobutyric acids in the BC99_M group. The enrichment of *Akkermansia*, a key commensal associated with a lean phenotype, likely contributed to improved intestinal barrier function via mucin degradation and stimulation of a healthier mucus layer [37]. Similarly, the increase in *Roseburia*, a major butyrate producer, may have enhanced butyrate-mediated anti-inflammatory and barrier-protective effects through GPR41/43 activation and HDAC inhibition [38]. Correlation analysis further revealed that *Roseburia* abundance was negatively correlated with serum LPS and TNF-α and positively correlated with IL-10, suggesting that BC99-mediated enrichment of these SCFA-producing bacteria is associated with enhanced intestinal SCFA bioavailability, an improved gut microenvironment, and suppressed systemic inflammation. Notably, a recent human trial on BC99 observed a similar phenomenon: in 66 overweight/obese adults, an 8-week intervention with BC99 (5 × 10^9^ CFU/day) significantly improved gut microbiota β-diversity, and weight loss was negatively correlated with an increase in the abundance of the genus *Parabacteroides* [35]. This suggests a cross-species consistency in the microbiota-modulating effects of BC99.

HFD-induced dysbiosis is often accompanied by downregulation of tight junction proteins, increased intestinal permeability, and LPS translocation into the bloodstream, triggering metabolic endotoxemia [39]. In the present study, the HFD group showed elevated serum levels of ZO-1 and occludin (indicating leakage), decreased expression of these proteins in colon tissue, and disrupted, discontinuous immunofluorescence signals. BC99 intervention significantly reversed these abnormalities, concurrently reducing serum levels of LPS and pro-inflammatory cytokines (TNF-α, IL-6, IL-1β) while elevating the anti-inflammatory cytokine IL-10. These findings align with a recent study showing that chitosan-stabilized selenium nanoparticles ameliorate HFD-induced NAFLD by upregulating ZO-1, occludin, and Muc2 expression, thickening the intestinal mucus layer, lowering LPS levels, and significantly increasing the abundance of *Akkermansia* and *Bifidobacterium* while reducing obesity-related genera like *Lachnoclostridium* [40]. This underscores that restoring intestinal barrier integrity is a critical step in blocking LPS-driven inflammatory cascades.

Untargeted metabolomics revealed that BC99 intervention significantly reshaped the serum lysophosphatidylcholine profile, restoring the levels of LysoPC 18:1, 18:2, and 20:4 that were decreased in the HFD group. LysoPCs are intermediates of membrane phospholipid metabolism generated by phospholipase A_2_ hydrolysis of phosphatidylcholine; they can also originate from the phospholipid metabolism of gut microbes [41]. Specific long-chain unsaturated LysoPCs (18:1, 18:2) possess anti-inflammatory and antioxidant properties and can act as endogenous ligands for PPARγ, promoting normal adipocyte differentiation and insulin sensitization. Recent research has provided direct evidence for the PPARγ-mediated anti-inflammatory effects of LysoPCs. A study demonstrated that carboxylesterase 2A knockout rats exhibited elevated hepatic LysoPCs and PCs, which activated PPARγ expression. Despite simple lipid accumulation, these rats showed significant protection against LPS- and diet-induced steatohepatitis [42]. Coupled with our findings, it is plausible that the LysoPC profile remodeling induced by BC99 contributes to the resolution of adipose tissue inflammation and the improvement in insulin sensitivity, potentially through PPARγ activation [43].

BC99 intervention also significantly altered the levels of the conjugated bile acids GUDCA and GDCA, and these changes were positively correlated with the abundances of *Lachnoclostridium* and *Akkermansia*. Certain strains within the genus *Lachnoclostridium* possess bile salt hydrolase (BSH) activity, which hydrolyzes the amide bond of conjugated bile acids, releasing free bile acids [44]. KEGG pathway enrichment analysis highlighted the “primary bile acid biosynthesis” pathway as significantly modulated, further supporting the involvement of bile acid metabolism remodeling in the mechanism of action of BC99. Bile acids function not only as fat emulsifiers but also as important signaling molecules that regulate glucose and lipid metabolism, energy expenditure, and immune responses by activating the nuclear receptor FXR and the membrane receptor TGR5 [45]. GUDCA, the glycine conjugate of ursodeoxycholic acid (UDCA), is generally considered an FXR antagonist [29]. Upon deconjugation, the resulting free UDCA can activate TGR5, promoting GLP-1 secretion from intestinal L-cells and thereby improving insulin release and energy utilization [46]. In states of obesity and diabetes, dysbiosis-induced alterations in the bile acid pool can disrupt the FXR/TGR5 signaling balance, exacerbating metabolic disturbances and chronic inflammation [47].

Synthesizing these findings, we propose a hypothetical model for the anti-obesity effects of BC99 in rats, based on the observed correlations. We hypothesize that daily ingestion of BC99 spores may lead to enrichment of beneficial bacterial groups, including short-chain fatty acid producers (e.g., *Roseburia*, *Lachnospiraceae*), mucin degraders (e.g., *Akkermansia*), and bacteria possessing bile salt hydrolase activity (e.g., *Lachnoclostridium*) [48,49]. These microbial shifts were associated with a cascade of metabolite changes: increased intestinal concentrations of SCFAs (butyrate, propionate), remodeling of the serum LysoPC profile towards a more anti-inflammatory and insulin-sensitive pattern, and enhanced deconjugation of the conjugated bile acids GUDCA and GDCA, altering the free-to-conjugated bile acid ratio [50]. These metabolites, in turn, activate key host signaling pathways—SCFAs exert anti-inflammatory and barrier-protective effects via GPR41/43 activation and HDAC inhibition; LysoPCs may improve adipose tissue function through PPARγ activation; and the altered bile acid pool modulates intestinal FXR and TGR5 signaling, influencing lipid metabolism and energy homeostasis [51,52]. This “microbiota–bile acid–host receptor” axis, acting in concert with the pathways mediated by SCFAs and LysoPCs, ultimately culminates in the restoration of the intestinal barrier, attenuation of endotoxemia, resolution of systemic inflammation, improvement in glucose and lipid metabolism, and reversal of the obese phenotype.

However, this study has certain limitations. First, although the correlation analyses indicate strong associations between gut microbiota, metabolite changes, and phenotypic improvements, causality has not been established. Therefore, while our results support the potential of BC99 as a candidate probiotic for obesity management, clinical trials are essential to confirm its efficacy, determine optimal dosing, and assess safety in human populations. Fecal microbiota transplantation (FMT) experiments, transplanting microbiota from BC99-treated donors into germ-free or antibiotic-treated mice, represent the gold standard for verifying whether the reshaped microbiota is sufficient to recapitulate the anti-obesity phenotype [53,54]. Second, the functional roles of key metabolites are currently inferred primarily from literature; future studies involving oral supplementation with specific metabolites or the use of metabolic inhibitors could provide direct in vivo validation [7]. Third, it should be noted that the metabolomic changes identified in this study, including LysoPCs and conjugated bile acids, were based on untargeted LC-MS analysis, which provides relative rather than absolute quantification. Moreover, the activity of related enzymes such as bile salt hydrolase (BSH) was not directly measured. Future studies employing targeted metabolomics with authentic standards, as well as enzymatic activity assays, are warranted to validate these findings and provide more precise mechanistic insights.

In summary, this study demonstrates that *W. coagulans* BC99 supplementation is associated with amelioration of HFD-induced obesity and related metabolic disorders in rats. The observed correlations between gut microbiota remodeling, metabolite modulation, and phenotypic improvements support a potential ‘microbiota–metabolite–host’ axis in this animal model. These findings suggest that the application of BC99 in clinical settings warrants further investigation.

## 5. Conclusions

This study demonstrated that BC99 ameliorates obesity in a rat model. BC99 intervention significantly reduced body weight gain, adipose accumulation, and hepatic steatosis, while improving lipid metabolism, glucose homeostasis, and intestinal barrier function. BC99 reshaped the gut microbiota by enriching beneficial genera such as *Akkermansia*, *Roseburia*, and *Lachnospiraceae_NK4A136_group*, accompanied by increased fecal short-chain fatty acids. Untargeted metabolomics further revealed that BC99 upregulated anti-inflammatory lysophosphatidylcholines (LysoPCs) and modulated conjugated bile acids (GUDCA, GDCA). These microbial and metabolic changes were closely associated with reduced systemic inflammation and restored gut integrity. Collectively, these findings provide multi-omics evidence supporting the potential of *W. coagulans* BC99 as a functional probiotic for obesity management.

## Data Availability

The original contributions presented in this study are included in the article/Supplementary Materials. Further inquiries can be directed to the corresponding author.

## Supplementary Materials

The following supporting information can be downloaded at: https://www.mdpi.com/article/10.3390/metabo16040228/s1, Figure S1: (A) OPLS-DA score plot showing the separation among the CON, HFD, and BC99_M groups. (B) OPLS−DA permutation test.
