# Supplementary material for: Weizmannia coagulans BC99 Ameliorates Obesity and Associated Inflammation by Remodeling the Gut Microbiota and Regulating Lysophosphatidylcholine and Conjugated Bile Acid Metabolism
Source: Metabolites. 2026 Mar 30;16(4):228. doi: 10.3390/metabo16040228 (PMC13117854; doi:10.3390/metabo16040228)
Supplement: Supplementary file 1 [file metabolites-16-00228-s001.zip › metabolites-4205130-supplementary.pdf]

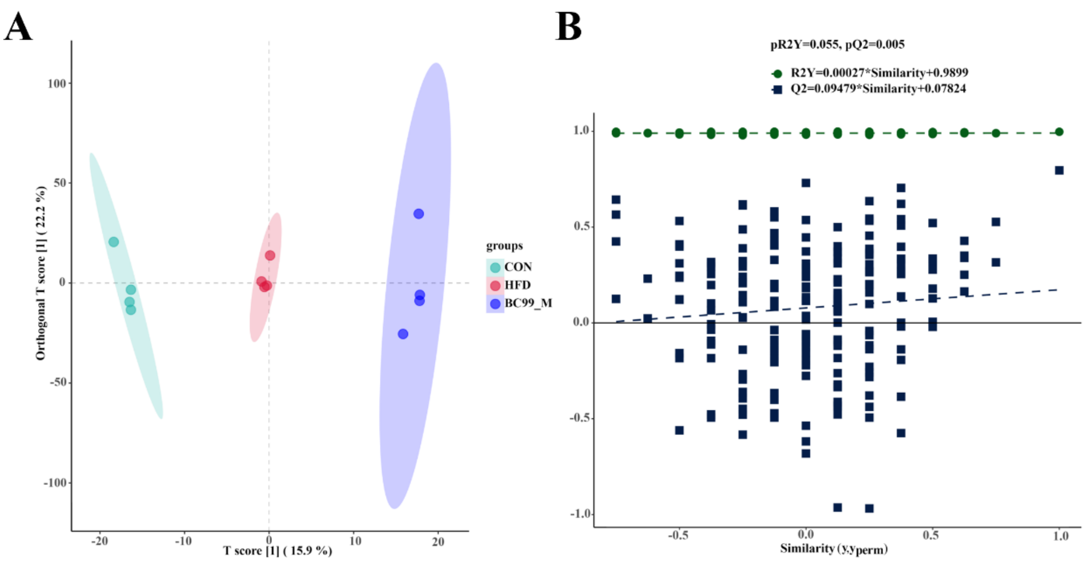

Figure S1. (A) OPLS-DA score plot showing the separation among the CON, HFD, and BC99_M groups. (B) OPLS–DA permutation test.
